# Demonstration of potential link between Helicobacter pylori related promoter CpG island methylation and telomere shortening in human gastric mucosa

**DOI:** 10.18632/oncotarget.9764

**Published:** 2016-06-01

**Authors:** Tomomitsu Tahara, Tomoyuki Shibata, Masaaki Okubo, Tomohiko Kawamura, Noriyuki Horiguchi, Takamitsu Ishizuka, Naoko Nakano, Mitsuo Nagasaka, Yoshihito Nakagawa, Naoki Ohmiya

**Affiliations:** ^1^ Department of Gastroenterology, Fujita Health University School of Medicine, Toyoake, Japan

**Keywords:** DNA methylation, telomere length, gastric mucosa, H. pylori, gastritis

## Abstract

**Background:**

Telomere length shortening in Helicobacter pylori (*H. pylori*) infected gastric mucosa constitutes the earliest steps toward neoplastic transformation. In addition to this genotoxic changes, epigenetic changes such as promoter CpG island (PCGI) methylation are frequently occurred in *H. pylori* infected gastric mucosa. The aim of this study was to investigate a potential link between *H. pylori* related PCGI methylation and telomere length shortening in the human gastric mucosa.

**Methods:**

Telomere length was measured in non-neoplastic gastric mucosa from 106 cancer-free subjects. To identify *H. pylori* related PCGI methylation, bisulfite pyrosequencing was used to quantify the methylation of 49 PCGIs from 47 genes and *LINE1* repetitive element

**Results:**

We identified five PCGIs (*IGF2*, *SLC16A12*, *SOX11*, *P2RX7* and *MYOD1*), which the methylation is closely associated with *H. pylori* infection. Hypermethylation of all these PCGIs was associated with development of pathological state from normal to mild, active, and atrophic gastritis (*P*<0.001) and lower pepsinogen I/II ratio (*P*<0.05), an indicator for gastric mucosal atrophy. Telomere shortening was significantly associated with mean Z score methylation of five PCGIs (R=−0.39, *P*<0.0001) and four of these locus (*IGF2*: R=−0.35, *P*=0.0003, *SLC16A12*: R=−0.35, *P*=0.0002, *P2RX7*: R=−0.29, *P*=0.003, and *MYOD1*: R=−0.33, *P*=0.0005). Multivariate analysis revealed that telomere shortening held an increased risk for hypermethylation (odds ratio: 1.71, 95% confidence interval: 1.11-2.63, *P*=0.016).

**Conclusion:**

Potential link between *H. pylori* related PCGI methylation and telomere shortening emphasize the importance of genotoxic-epigenetic interaction in the pathological state of *H. pylori* infected gastric mucosa.

## INTRODUCTION

Telomeres consist of repetitive nucleotide sequences and an associated terminal protein complex that help avoid loss of chromosomal integrity [1]. Telomere shortening in genomic DNA seems to reflect lifetime accumulative oxidative stress through environmental exposures [2–6]. Shortened telomere lengths were also observed in human epithelial cancers due to the formation of complex nonreciprocal translocations and increased chromosome instability [7–10]. Telomere length abnormalities also occur early in epithelial carcinogenesis, suggesting that telomere length may serve as a useful biomarker in human cancers.

Helicobacter pylori (*H. pylori*) infected gastric mucosa is characterized as chronic inflammation [11, 12], which is closely linked with telomere length shortening [13, 14]. These facts suggest that shortened telomere constitutes the earliest steps toward neoplastic transformation relevant to gastric cancer predisposition. In addition to this genotoxic changes, epigenetic alterations may also play a role in this process. For example, regional promoter CpG island (PCGI) hypermethylation and global DNA hypo methylation are frequently occurred in *H. pylori* infected gastric mucosa in relation to severity of inflammation and/or atrophy [15, 16]. Our preliminary result showed that telomere shortening correlated with DNA methylation at *CDH1* gene promoter [17]. Induction of methylation in the gastric mucosa by *H. pylori* involves complex biological processes that are still not completely understood. The aim of this study was to investigate a potential link between *H. pylori* related PCGI hypermethylation and telomere length shortening in the human gastric mucosa.

## RESULTS

### Identification of hyper methylated PCGIs in *H. pylori* positive gastric mucosa and their relationship with severity of *H. pylori* related gastritis

**Figure 1:**
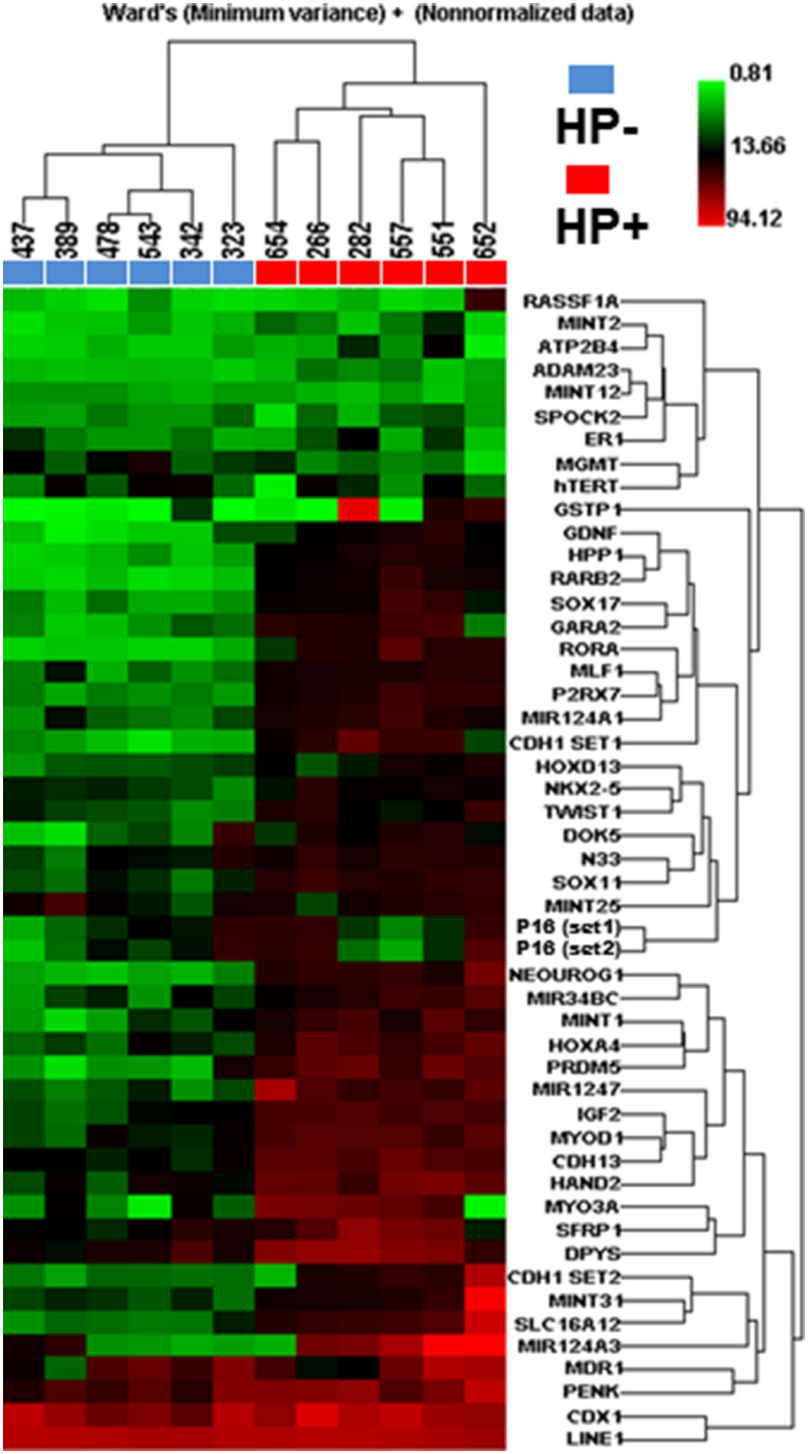
Unsupervised hierarchical clustering analysis of 49 PCGIs and *LINE1* repetitive element among six *H. pylori* negative (blue boxes) and six positive cases (red boxes) Numbers listed above the blue and red boxes indicates sample ID.

**Figure 2:**
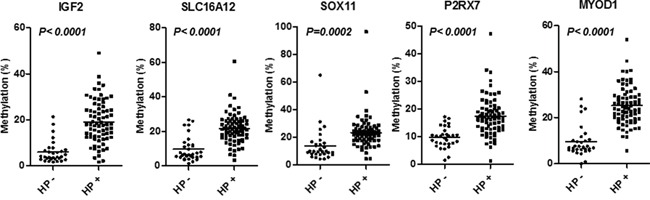
Methylation status of five PCGIs (*IGF2*, *SLC16A12*, *SOX11*, *P2RX7* and *MYOD1*) among all subjects with or without *H. pylori* infection Horizontal bars represents mean methylation percentage. The statistical analysis was performed using Student's t-Test.

Age and sex distributions, *H. pylori* infection status, prevalence of peptic ulcer disease and status of histological gastritis are shown in Table 1. To explore hyper methylated PCGIs in *H. pylori* positive gastric mucosa, we initially performed unsupervised hierarchical clustering analysis of 49 PCGIs and *LINE1* among six *H. pylori* negative and six positive cases (Figure 1). This analysis showed that all *H. pylori* positive gastric mucosa was clearly clustered as hyper methylated samples compared with the *H. pylori* negative mucosa. Based on this result, we selected five candidate PCGIs (*IGF2*, *SLC16A12*, *SOX11*, *P2RX7* and *MYOD1*) and *LINE1* repetitive element for further analysis. As expected, hypermethylation of all five PCGIs were tightly linked to *H. pylori* infection status (all *P*<0.0001, Figure 2). In line with previous result showing correlation between DNA methylation and *H. pylori* related gastritis [16], development of pathological state from normal to mild, active, and atrophic gastritis was correlated with hypermethylation of all these PCGIs (all *P*<0.001, Figure S1). We also showed that lower PG I/II ratio that indicates the severity of gastric mucosal atrophy was significantly associated with hypermethylation of all these PCGIs (all *P*<0.05, Figure S2).

**Table 1:** Clinic-pathological features of subjects

Variables	
Total number of subjects: n	106
Mean age: +/−SD	59.4+/−13.1
Gender: male n (%)	64 (60.4%)
*H. pylori* positives: n (%)	75 (70.8%)
Gastric ulcer: n (%)	15 (14.2%)
Duodenal ulcer: n (%)	12 (11.3%)
Normal gastric mucosa: n (%)	15 (14.2%)
Mild gastritis: n (%)	21 (19.8%)
Active gastritis: n (%)	24 (22.6%)
Atrophic gastritis: n (%)	46 (43.4%)

We also observed that Methylation of three PCGIs (*SLC16A12*, *SOX11* and *MYOD1*) were also associated with increasing age (all *P*<0.05, Figure S3), while this association seemed smaller compared to the associations between methylation status and *H. pylori* infection, degree of histological gastritis and PG I/II ratio.

### Telomere shortening in *H. pylori* related gastritis and its relationship with PCGI methylation

**Figure 3:**
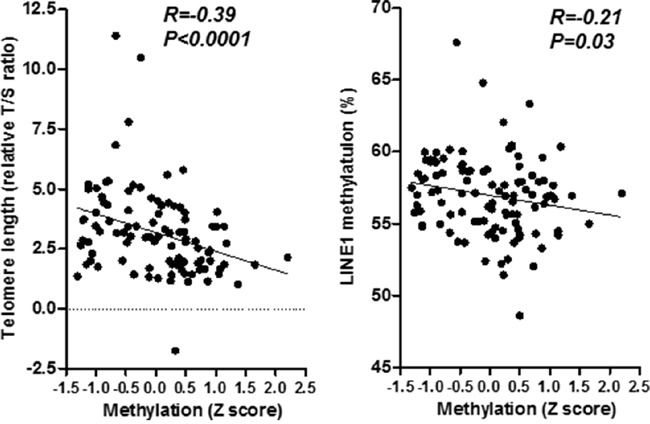
Association between telomere shortening and mean Z score methylation of five PCGIs (left) and methylation of *LINE 1* repetitive element (right) Statistical analysis was performed using the Spearman correlation analysis.

**Figure 4:**
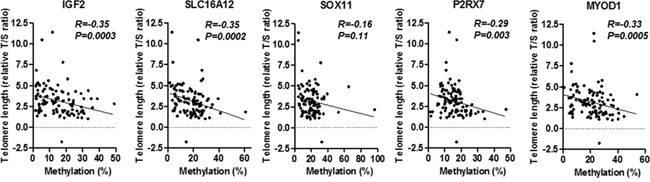
Association between telomere shortening and methylation status of five PCGIs (*IGF2*, *SLC16A12*, *SOX11*, *P2RX7* and *MYOD1*) Statistical analysis was performed using the Spearman correlation analysis

Telomere length was significantly correlated with *H. pylori* infection and histological gastritis (Figure S4), which is in line with our resent study [17]. We found that telomere shortening was significantly associated with mean Z score methylation of selected five PCGIs (R=−0.39, *P*<0.0001, Figure 3). In detail, methylation of four out of all five locus significantly associated with telomere shortening (*IGF2*: R=−0.35, *P*=0.0003, *SLC16A12*: R=−0.35, *P*=0.0002, *P2RX7*: R=−0.29, *P*=0.003, and *MYOD1*: R=−0.33, *P*=0.0005, Figure 4). *LINE1* repetitive element, an indicator for genome wide hypo methylation has been shown to be associated with *H. pylori* related enlarged fold gastritis and PCGI methylation [25]. We also found significant association between *LINE1* hypo methylation and mean Z score methylation of all these PCGIs (R=−0.21, *P*=0.03, Figure 3) as well as three out of all five locus (*IGF2*: R=−0.26, *P*=0.008, *SOX11*: R=−0.19, *P*=0.05, and *MYOD1*: R=−0.21, *P*=0.03, Figure S5) but this association seemed to be marginal when compared to the association with telomere shortening (Figure 3 and 4). To assess the factors related to DNA methylation in the gastric mucosa, univariate and multivariate analysis were performed. Since the mean Z score methylation of all five PCGIs in the gastric mucosa presented an approximately Gaussian distribution, with over representation of methylation-high cases (data not shown), we set cut-off values of 0.3 (mean Z score methylation) for the definition of methylation-high cases. Univariate analysis revealed that age (odds ratio: 1.04, 95% confidence interval: 1.00-1.07, *P*=0.04), *H. pylori* positive (odds ratio: 34.3, 95% confidence interval: 4.44-264.62, *P*=0.0007), duodenal ulcer (odds ratio: 3.70, 95% confidence interval: 1.04-13.20, *P*=0.04), atrophic gastritis (odds ratio: 4.67, 95% confidence interval: 2.02-10.80, *P*=0.0003) and telomere length shortening (odds ratio: 1.96, 95% confidence interval: 1.35-2.84, *P*=0.0004) were significantly associated with methylation-high. (Table 2) Multivariate analysis of these factors revealed that age (odds ratio: 1.05, 95% confidence interval: 1.01-1.09, *P*=0.03), *H. pylori* positive (odds ratio: 15.26, 95% confidence interval: 1.78-130.97, *P*=0.013), duodenal ulcer (odds ratio: 7.68, 95% confidence interval: 1.43-41.20, *P*=0.0174) were significantly associated with methylation-high. We observed that telomere length shortening was also remained as an independent factor associated with methylation-high (odds ratio: 1.71, 95% confidence interval: 1.11-2.63, *P*=0.016). (Table 3)

**Table 2:** Univariate analysis assessing the factors related to the methylation high

Variables	Odds ratio (95% confidence interval)	P value
Age	1.04 (1.00-1.07)	0.04
Gender (male)	0.88 (0.40-1.96)	0.76
*H. pylori* positive	34.3 (4.44-264.62)	0.0007
Gastric ulcer	2.01 (0.67-6.04)	0.21
Duodenal ulcer	3.70 (1.04-13.20)	0.04
Active gastritis	1.47 (0.58-3.68)	0.41
Atrophic gastritis	4.67 (2.02-10.80)	0.0003
Telomere shortening	1.96 (1.35-2.84)	0.0004
*LINE1* methylation	1.09 (0.94-1.27)	0.24

* Methylation high, mean Z score of methylation>0.3.

**Table 3:** Multivariate analysis assessing the factors related to the methylation high

Variables	Odds ratio (95% confidence interval)	P value
Age	1.05 (1.01-1.09)	0.03
*H. pylori* positive	15.26 (1.78-130.97)	0.013
Duodenal ulcer	7.68 (1.43-41.20)	0.017
Atrophic gastritis: n (%)	2.08 (0.70-6.20)	0.19
Telomere shortening	1.71 (1.11-2.63)	0.016

* Methylation high, mean Z score of methylation>0.3.

## DISCUSSION

Although the mechanisms of methylation induction in the gastric mucosa by *H. pylori* are not completely understood, chronic inflammation, pro-inflammatory cytokines and oxidative stress may have a role in this process [26–28]. We have selected five PCGIs (*IGF2*, *SLC16A12*, *SOX11*, *P2RX7* and *MYOD1*) from cancer and inflammation/age related panels. Methylation of these genes well correlated with *H. pylori* infection, histological and serological gastritis, which is consistent with previous studies [15, 16]. We showed that these PCGI hypermethylation well correlated with telomere shortening. Hypermethylation of all individual locus as well as their mean Z score was closely associated with telomere shortening. Multivariate analysis demonstrated that telomere length shortening is an independent factor associated with PCGI hypermethylation in the gastric mucosa. Our result emphasize the importance of genotoxic-epigenetic interaction in the pathological state of *H. pylori* infected gastric mucosa. Telomere shortening was identified in inflammatory tissues such as *H. pylor*i–positive gastric epithelial tissue and colonocytes in ulcerative colitis [13, 14, 17, 29], while it is also observed in aged peripheral leukocyte DNA. Thus, it seems telomeres shortening is a consequence of cell replication and oxidative damage [30]. There have been report showing potential link between telomere shortening and DNA methylation in other cell types. Telomere shortening is correlated with promoter methylation profile of p16/Rb and p53/p21 pathways in breast cancer tissue [31]. In blood cells, biological age predicted by DNA methylation status rather than chronological age is well correlated more with telomere length shortening and acquired aplastic anemia or dyskeratosis congenita - two diseases associated with progressive bone marrow failure [32]. The link between telomere shortening and subtelomeric DNA methylation was also confirmed in vitro using human fibroblasts cultured under conditions of chronic oxidative stress, induced by tert-butyl hydroperoxide [33]. Together with link between *H. pylor*i related PCGI methylation and telomere shortening in the stomach, it is suggested that alteration of telomere shortening and DNA methylation predict various pathological state and clinical outcomes across the different tissue types. Since *H. pylori* infected gastric mucosa enhances chronic inflammation and increased cell turnover, it is reasonable to expect the relationship between PCGI hypermethylation and telomere shortening. On the other hand, direct mechanistic link of PCGI hypermethylation and telomere shortening has not been clearly demonstrated so far [34, 35]. Further investigation will be needed to clarify this issue.

*LINE1* repetitive element, an indicator for genome wide hypo methylation has been shown to be associated with *H. pylori* related enlarged fold gastritis, which is a form of gastritis with an increased risk of gastric cancer [25]. The *LINE1* hypo methylation is also associated with PCGI hypermethylation in certain genes, such as *CDH1*, *CDH13*, and *PGP9.5* genes [25]. We also found significant association between *LINE1* hypo methylation and CpG island promoter methylation but this association seemed to be marginal compared to the association with telomere shortening. One merit in our study is we carefully selected PCGI methylation closely linked to *H. pylori* infection. The result suggest that *H. pylori* related CpG island promoter methylation is correlated more with telomere shortening than genome wide hypo methylation. Since telomere shortening and increased PCGI hypermethylation in *H. pylori* infected gastric mucosa is likely to be an outcome of chronic inflammation or increased cell turnover, *H. pylori* related gastritis can be therefore viewed as premature aging of gastric epithelial cells. Chronic inflammation in *H. pylori* infected gastric mucosa leads to telomere shortening and increased PCGI hypermethylation, which may eventually lead to cancer progression. The potential usefulness of telomere shortening and related CpG island hypermethylation as the molecular marker for gastric cancer needs to be evaluated in various clinical settings. Recent comprehensive genome wide methylation analysis identified PCGIs at which methylation levels are associated with leukocyte telomere length. The result demonstrated that the associated sites were enriched in subtelomeric loci (within 4 Mb of the telomere), and also at loci in imprinted regions [36]. Further investigations to elucidate the relationship between telomere length and DNA methylation should be needed to understand the biological importance of genotoxic-epigenetic interaction in pathological state of human gastric mucosa.

## MATERIALS AND METHODS

### Subjects

Enrolled were 106 subjects who were attending the Endoscopy Center of Fujita Health University Hospital between January 2005 and April 2010. All subjects performed upper gastroscopy for various indications such as health check, secondary complete check-up of stomach cancer following to barium X-ray examination, follow-up examination of ulcer diseases, or for the complaint of abdominal discomfort. Patients who had severe systemic disease, malignancy in the stomach or other organ, previous history of *H. pylori* eradication as well as continuous usage of non-steroidal anti-inflammatory drugs were not included. The cohort was partly recruited from previous studies investigating the genetic polymorphisms, and telomere length among gastroduodenal diseases [17–19]. The Ethics Committee of the Fujita Health University School of Medicine approved the protocol, and prior, written informed consent was obtained from all participating subjects.

### DNA extraction, and detection of *H. pylori* infection, histological assessment

During upper gastroscopy, biopsy specimens were taken from the antrum and the upper corpus along the greater curvature from grossly non-pathological mucosa in all patients. The specimens taken from the antrum were cut into two pieces, and one part of specimens was immediately frozen and stored at −80°C until for the molecular experiment. Genomic DNA was extracted directly from these frozen specimens using the standard protein precipitating method. Other specimens were fixed in 10% buffered formalin and embedded in paraffin for microscopic histological examination. Using hematoxylin and eosin-stained histological slides, the extent of chronic inflammation, atrophy, and metaplasia in the antrum were assessed according to the updated Sydney system [20], with each factor being scored from 0 to 3 (0, normal; 1, mild; 2, moderate; 3, severe). This assessment was performed by senior pathologists in our hospital who were blinded to the characteristics of subjects. Based on this scoring system, subjects were divided in to following four categories; Normal, all 0; Active gastritis, 2 or higher score of acute or chronic inflammation; Atrophic gastritis, 2 or higher score of acute or chronic inflammation; Mild gastritis, all others. Histological assessment also confirmed that all the specimens contained more than 70% of epithelial cells. Assessment of *H. pylori* infection status was performed using two biopsy specimens taken from the antrum and the upper corpus by immunohistochemistry using the polyclonal rabbit anti-Helicobacter pylori antibody (FLEX Anti-Helicobacter Pylori, Code GA523, Dako, Tokyo, Japan).

### Serological evaluation

The serum pepsinogen (PG) I/II ratio was calculated based on the data of the serum PG I and PG II levels measured by radioimmunoassay in 80 cases. PG I/II ratio that showed a decrease in proportion to the severity of gastric mucosal atrophy [21 22] was used as a marker of atrophic gastritis.

### Relative average telomere length measurement

Relative telomere length was measured as a comparative quantification, in particular as abundance of telomeric template vs. a single-copy gene (T/S) by quantitative real-time PCR as described previously. [23] For the quantitative real-time PCR, the iTaq SYBR Green Supermix (Bio-Rad) and ABI Prism 7900HT Real-Time PCR System (Applied Biosystems) were used. The primers for telomeres and single-copy genes (h-globin) are listed in Supplementary Table 2. Detailed protocol of experiment is available in our recent study [17].

### Selection of candidate panels and PCGI methylation analysis by bisulfite pyrosequencing

Bisulfite pyrosequencing was used to quantify the methylation of 49 PCGIs from 47 genes. We reasoned that there might be two approaches to selecting genes (all selected from the literature), one based on frequency of methylation in gastric and other cancers (*MINT1*, *MINT2*, *MINT12*, *MINT25*, *MINT31*, *RORA*, *GDNF*, *PRDM5*, *MLF1*, *CDH1*, *p16* etc.) and a separate one based on methylation in non-neoplastic inflamed/aged tissues (*ER*, *CDH13*, *MYOD1*, *SFRP1*, *P2RX7*, *IGF2*, *N33*, *MIR124A1*, *MIR124A3* etc.) [24]. We also evaluated the methylation status of the *LINE1* repetitive element using bisulfite pyrosequencing. Bisulfite-treated genomic DNA was used to evaluate the methylation status by the bisulfite pyrosequencing. Bisulfite treatment of DNA was carried out with BislFast DNA Modification Kit (Toyobo, Osaka, Japan) according to the manufacturer's protocol. Pyrosequencing was carried out using PSQ24 system with Pyro-Gold reagent Kit (QIAGEN, Tokyo, Japan), and the results were analyzed using PyroMark Q24 software (QIAGEN). The primers used for pyrosequencing are listed in Supplementary Table S1.

### Data analysis

Using 12 gastric mucosa derived from six *H. pylori* negative and six positive patients, an unsupervised hierarchical clustering analysis was used to identify distinct subgroups based on the methylation status of 49 PCGIs and *LINE1* repetitive element. Continuous variables between two groups was assessed using the Student's t-Test. Continuous variables among more than two groups were assessed using the One way ANOVA. The correlation of continuous variables between two groups was assessed using the Spearman correlation analysis. Univariate and multivariate analysis were also performed to assess the factors related to PCGI methylation. P value <0.05 was considered statistically significant.

## SUPPLEMENTARY FIGURES AND TABLES




