# Supplementary material for: Demonstration of potential link between Helicobacter pylori related promoter CpG island methylation and telomere shortening in human gastric mucosa
Source: Oncotarget. 2016 Jun 1;7(28):43989–96. doi: 10.18632/oncotarget.9764 (PMC5190073; doi:10.18632/oncotarget.9764)
Supplement: Supplementary file 1 [file oncotarget-07-43989-s001.pdf]

Demonstration of potential link between Helicobacter pylori related promoter CpG island methylation and telomere shortening in human gastric mucosa

SUPPLEMENTARY FIGURES AND TABLES

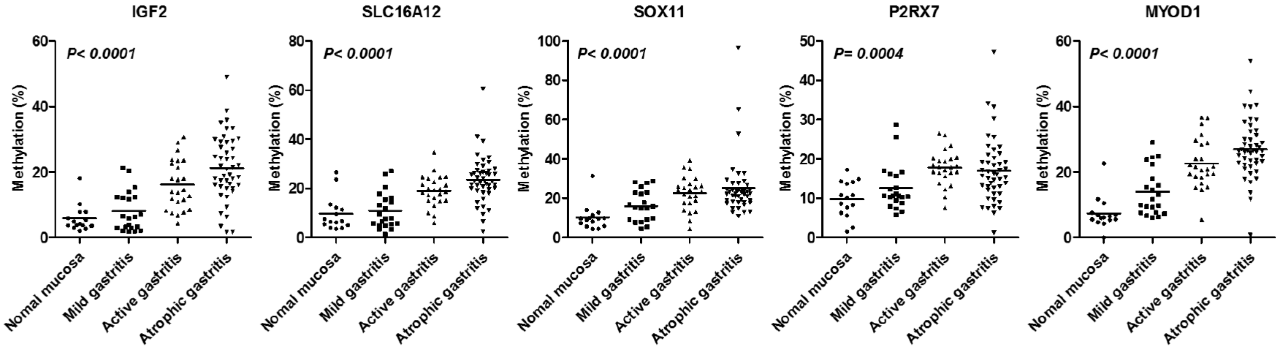

Supplementary Figure S1: Association between methylation status of five PCGIs (*IGF2*, *SLC16A12*, *SOX11*, P2RX7 and *MYOD1*) and development of pathological state of *H. pylori* related gastritis. The statistical analysis was performed using one way ANOVA.

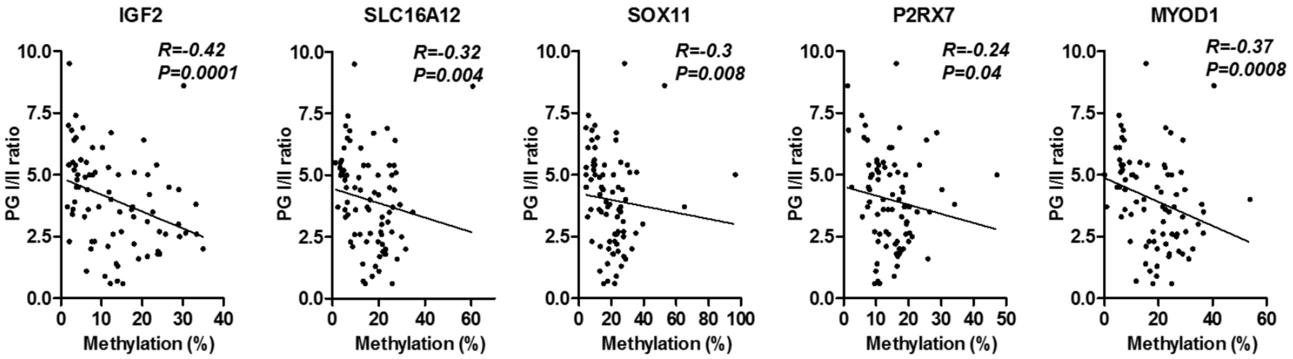

Supplementary Figure S2: Association between methylation status of five PCGIs (*IGF2*, *SLC16A12*, *SOX11*, *P2RX7* and *MYOD1*) and serum pepsinogen (PG) I/II ratio. Statistical analysis was performed using the Spearman correlation analysis.

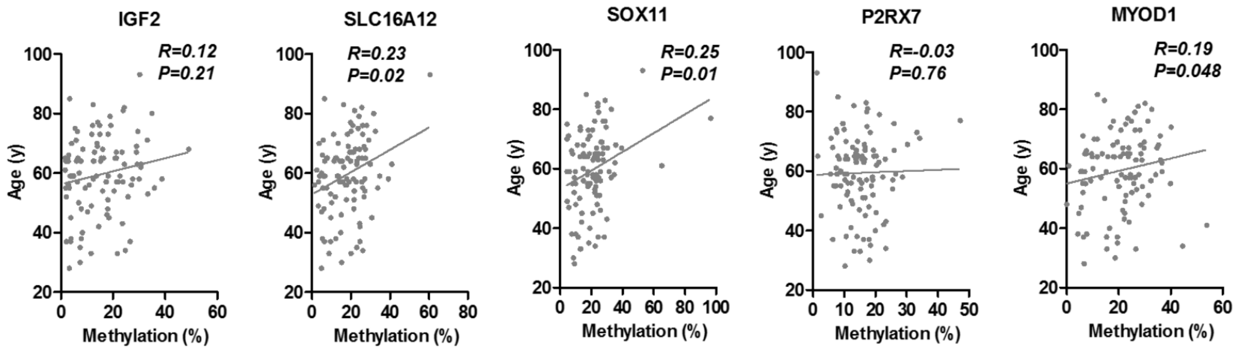

Supplementary Figure S3: Association between methylation status of five PCGIs (*IGF2*, *SLC16A12*, *SOX11*, *P2RX7* and *MYOD1*) and aging. Statistical analysis was performed using the Spearman correlation analysis.

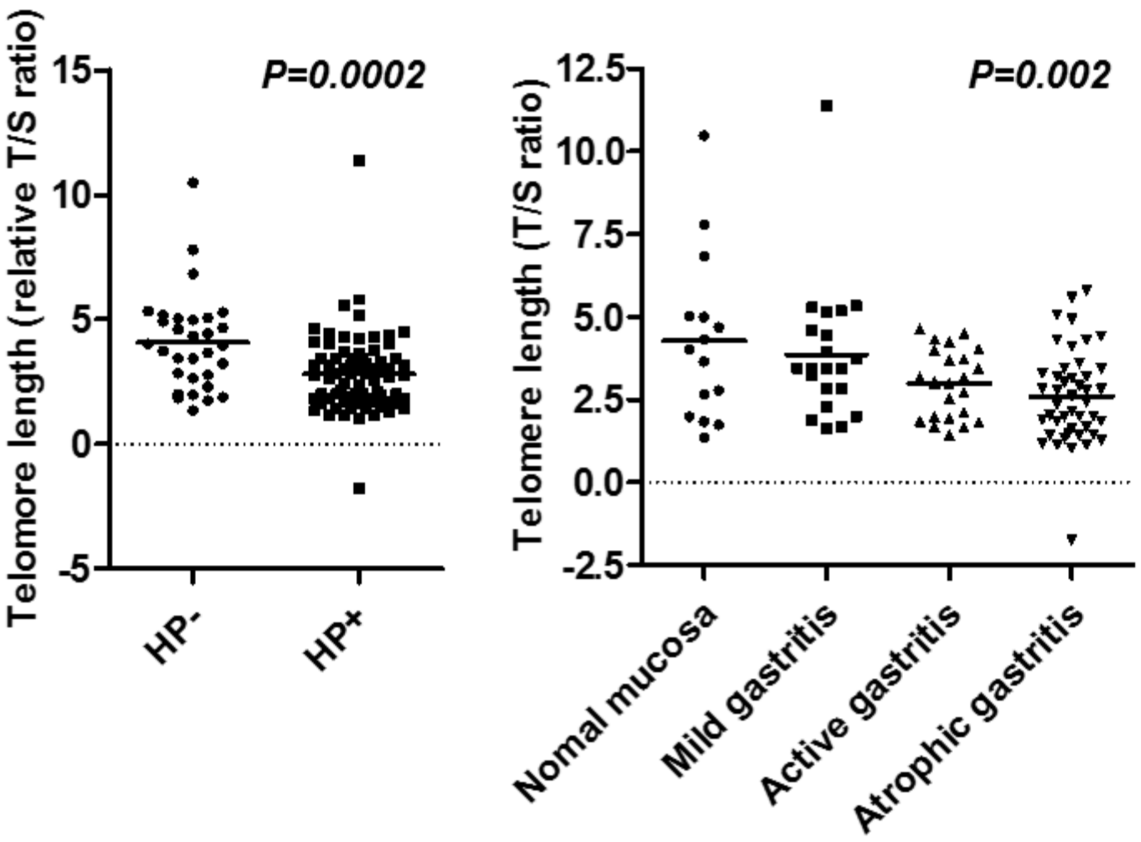

Supplementary Figure S4: Association between telomere shortening and *H. pylori* infection (left) and development of pathological state of *H. pylori* related gastritis (right). The statistical analysis was performed using Student's t-Test (left) and one way ANOVA (right), respectively.

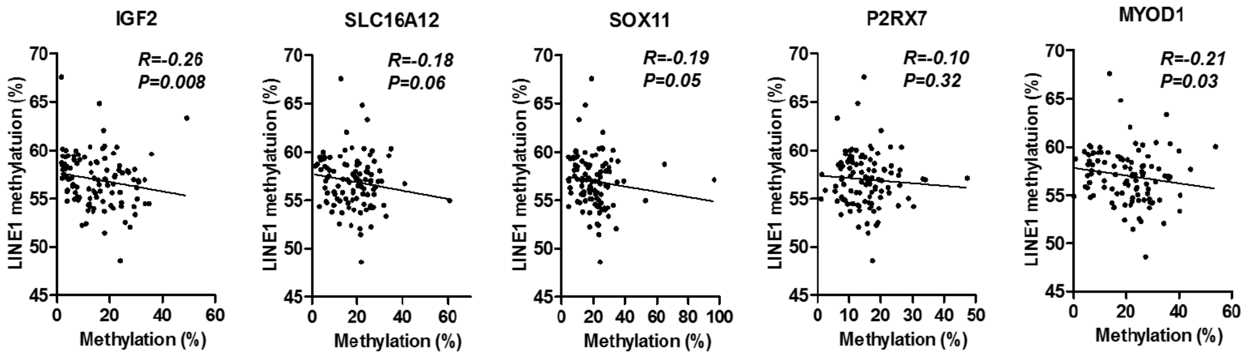

Supplementary Figure S5: Association between methylation status of five PCGIs (*IGF2*, *SLC16A12*, *SOX11*, P2RX7 and *MYOD1*) and *LINE1* repetitive element. Statistical analysis was performed using the Spearman correlation analysis.

Supplementary Table S1: Primer sequences used in pyrosequencing

See Supplementary File 1

Supplementary Table S2: Primer sequences used in telomere length measurement

Assay name	Forward primer	Reverse primer
	sequence	sequence
Telomere	CGGTTTGTTTGGGTTTGGGTT TGGGTTTGGGTTTGGGTT	GGCTTGCCTTACCCTTACCCTTA CCCTTACCCTTACCCT
Beta-globin	GCTTCTGACACAACTGTGTTCACTAGC	CACCAACTTCATCCACGTTCACC
