# Supplementary material for: Demonstration of potential link between Helicobacter pylori related promoter CpG island methylation and telomere shortening in human gastric mucosa
Source: Oncotarget. 2016 Jun 1;7(28):43989–96. doi: 10.18632/oncotarget.9764 (PMC5190073; doi:10.18632/oncotarget.9764)
Supplement: Supplementary file 2 [file oncotarget-07-43989-s002.doc]

**Supplementary Table 1**

| **Supplementary Table 1.** Primer sequences used in pyrosequencing | |  |  |  |  |
| --- | --- | --- | --- | --- | --- |
| Assay name | Forward primer  (1st step PCR) | Reverse primer  (1st step PCR) | Forward primer  (2nd step PCR) | Reverse primer  (2nd step PCR) | Sequencing primer |
|  | sequence | sequence | sequence | sequence | sequence |
| MINT1 | GGGTTGGAGAGTAGGGGAGTT | CCATCTAAAATTACCTCRATAACTTA | GGTTTTTTGTTAGYGTTTGTATTT | U-ATTAATCCCTCTCCCCTCTAAACTT | TTTAGTAAAAATTTTTTGGG |
| MINT2 | GGAAAGTGTTAGAAAAATGTGTTGTAT | CCTTCCTACAATTAAACATCAATTATAT | GGAAAGTGTTAGAAAAATGTGTTGTAT | U-CCTTCCTACAATTAAACATCAATTATAT | GATTTTGGATTTTGTTAAAG |
| MINT12 | GGATTAGGTTTTAGGGTTTAGAAG | AACCAAAACCATATCTAAATCAC | GGATTAGGTTTTAGGGTTTAGAAG | U-AACCAAAACCATATCTAAATCAC | GTTTTTTAGTAGATTGTGTT |
| MINT25 | TGTTTGTAAAGGGTTGGAATTATT | CCCRCCAAAACAACTTTA | TGTTTGTAAAGGGTTGGAATTATT | U-CCCRCCAAAACAACTTTA | TAGTTTATTATTTTTAAGAG |
| MINT31 | TGGTTTTAGTAAAGTGAGGG | AACCTAATAAATCACTCAATTC | TGGTTTTAGTAAAGTGAGGG | U-AACCTAATAAATCACTCAATTC | TGGTGATGGAGGTTATT |
| RORA | TTTGGTATTATAGAGTTGTTTTGAAAATAGAA | ACCCAAACTAACTCCATATTTTTTCC | TTTGGTATTATAGAGTTGTTTTGAAAATAGAA | U-ACCCAAACTAACTCCATATTTTTTCC | TGAAAATAGAAGATAGAGGGA |
| GDNF | AGGATTGAGAATTTTTGTTTTTGATT | CCAAACCCTAAATTAAACATTAACTCCA | AGGATTGAGAATTTTTGTTTTTGATT | U-CCAAACCCTAAATTAAACATTAACTCCA | TTTTGTTTTTGATTTGTTG |
| PRDM5 | TGAGGTTTTGGGGTTAGTTT | CRAATCCRTTCCTACCATTC | TGAGGTTTTGGGGTTAGTTT | U-CRAATCCRTTCCTACCATTC | GTTAATTTYGGGTTAATTAG |
| MLF1 | GGTGAAGTTATAGAGTAATGTTTAATGGGAAAGTA | CCCCACAAAAACTAACCTCCAAT | GGTGAAGTTATAGAGTAATGTTTAATGGGAAAGTA | U-CCCCACAAAAACTAACCTCCAAT | GTAGTTAGGTGTTAAGTATTTTTTAT |
| IGF2 | GAGGATTAGGGAGGGAAATATAGT | CCCAAACCCCCAAATTATC | GAGGATTAGGGAGGGAAATATAGT | U-CCCAAACCCCCAAATTATC | AATGGTTATTTAGTTTTTAG |
| DPYS | TTAGTTTTTTAGGGGGGAGGAGT | ACCCCCCAACTCTACCTCAAAC | TTAGTTTTTTAGGGGGGAGGAGT | U-ACCCCCCAACTCTACCTCAAAC | GTAGTATATGAGGTTAGGTTATAAAT |
| NKX2-5 | AGAGTAGGGTTGGGGAATATGG | CCCTCTCCTACCCCTTATACTCAA | AGAGTAGGGTTGGGGAATATGG | U-CCCTCTCCTACCCCTTATACTCAA | TAGGTGGGAGGTAGAA |
| CDH1 (set1) | GGAATTGTAAAGTATTTGTGAGTTTG | CAAATACCTACAACAACAACAACAAC | GGAATTGTAAAGTATTTGTGAGTTTG | U-CAAATACCTACAACAACAACAACAAC | GGAAGTTAGTTTAGATTTTA |
| CDH1(set2) | TTTGATTTTAGGTTTTAGTGAGT | ACCACAACCAATCAACAAC | TTTGATTTTAGGTTTTAGTGAGT | U-ACCACAACCAATCAACAAC | TAGTAATTTTAGGTTAGAGG |
| N33 | GYGYGTGGAGGAGATATTGTTT | CATTCTACCTCCTTTTTCTTCTAT | GYGYGTGGAGGAGATATTGTTT | U-CATTCTACCTCCTTTTTCTTCTAT | GTTTTTTTAAGTAGTAGGTAAG |
| PENK | GGAAAAGAGTAGGGTGTTTTAGGT | CCCCCAAAAATACTCCTTTCT | GGAAAAGAGTAGGGTGTTTTAGGT | U-CCCCCAAAAATACTCCTTTCT | GAGTAGGGTGTTTTAGGTAGT |
| SOX11 | AGTTGGGGGAGTGATGTTATTTA | AACAACCCCAAACCCCTCTCT | GGGGGAGTGATGTTATTTATATGAT | U-CCCCCAACTCTCCCAAAC | GAGATTTTAATTTTTTGTAGAAG |
| MYO3A | TTTTAGAGGGGAGGGTAGGGGTAGT | TCCACTCCATTAACCCAAATCAA | GGGGTAGTAGAGTAGGGGAAGAAT | U-TCCACTCCATTAACCCAAATCAA | AGAATTGGGTAGTTTGTAGA |
| MYOD1 | AATTAGGGGATAGAGGAGTATTGAAAG | ACAACCCTAAACRACTACACTTAACTC | GAAAGTTAGTTTAGAGGTGA | U-ACAACCCTAAACRACTACACTTAACTC | GAGGTTTGGAAAGGG |
| LINE1 | TTTTGAGTTAGGTGTGGGATATA | AAAATCAAAAAATTCCCTTTC | TTTTGAGTTAGGTGTGGGATATA | U-AAAATCAAAAAATTCCCTTTC | GGGTGGGAGTGAT |
| ADAM23 | AGGGAGTYGTAGTTGGGGAGGTTTTAA | TAAACRACRCCCCCTTTCC | AGGGAGTYGTAGTTGGGGAGGTTTTAA | U-TAAACRACRCCCCCTTTCC | TTGGGGAGGTTTTAAGT |
| DOK5 | GGAAAGGGTAGGGGTTGAA | TCCCCCACAACCTACTAAAAT | GGAAAGGGTAGGGGTTGAA | U-TCCCCCACAACCTACTAAAAT | TTTTTTTTTTAAAATAAGTTATTG |
| ER1 | TGTGTTTTTTTTTTAGGTGG | AACCATCCCAAATACTTTAATA | TGTGTTTTTTTTTTAGGTGG | U-AACCATCCCAAATACTTTAATA | GGATACGGTTTGTATTTTG |
| MDR1 | TAATTAGGGTAGGGAGTAGTTATTTGTG | CCAATAAACTCAAACTTCCTATAACA | TAATTAGGGTAGGGAGTAGTTATTTGTG | U-CCAATAAACTCAAACTTCCTATAACA | ATAGGAAGTTTGAGTTTATT |
| RARB2 | AGTTGGGTTATTTGAAGGTTA | TACCCAAACAAACCCTACTC | AAGTAGTAGGAAGTGAGTTGTTTAGA | U-CCCAAACAAACCCTACTC | TTTGAGGATTGGGATG |
| GSTP1 | AAGGAGGTTAGGGGTAAAAGTTATA | CCAAAACCTCCCCAATAC | GAGTTAGGGGGAGGATGT | U-CCAAAACCTCCCCAATAC | GGGGGAGGATGTTAAG |
| RASSF1A | GGGGGAGTTTGAGTTTATTGA | CTACCCCTTAACTACCCCTTCC | GGGGGAGTTTGAGTTTATTGA | U-CTACCCCTTAACTACCCCTTCC | GGGTAGTATTAGGTTGGAG |
| SLC16A12 | TAGAGGGAGAGGTGGTTTAGGTGAT | CACCCAAATTAAAATCCCAAACTC | TAGAGGGAGAGGTGGTTTAGGTGAT | U-CACCCAAATTAAAATCCCAAACTC | AAGGGTATTTTTTAAGGAAG |
| HOXD13 | TAGGAGTGGGTGGGTTTAGT | CCCAAAACCTACCATTAACTAC | TAGGAGTGGGTGGGTTTAGT | U-CCCAAAACCTACCATTAACTAC | TTAGGTTTTTTAGAGTTTTTATT |
| P16 (set1) | AATTTGGTAGTTAGGAAGGTTGTA | CCCCCCTACCAACAAAAA | AATTTGGTAGTTAGGAAGGTTGTA | U-CCCCCCTACCAACAAAAA | GTATAGAGGAGGAAGGAAA |
| P16 (set2) | AATTTGGTAGTTAGGAAGGTTGTA | CCCCCCTACCAACAAAAA | AATTTGGTAGTTAGGAAGGTTGTA | U-CCCCCCTACCAACAAAAA | GGATTTTTTTTTAATAGAGTGA |
| NEOUROG1 | TTTGGAGAAGTTTTGGTTAGTTTAGTT | ACCCCCCAATATTTACATAATTTATACTC | TTTGGAGAAGTTTTGGTTAGTTTAGTT | U-ACCCCCCAATATTTACATAATTTATACTC | GAGAAGTTTTGGTTAGTTTA |
| P2RX7 | GAAGAGTAGAGTTTTGGTTTAGTTT | AAACCACTTAATAATACCATAATTC | GAAGAGTAGAGTTTTGGTTTAGTTT | U-AAACCACTTAATAATACCATAATTC | TGTAGTGATGTTTTTTAGTATGAG |
| HPP1 | AGTTATTGGAGATTAAAATTTTATTAGG | AACTCTACCCCTTTCTTTCATTC | AGTTATTGGAGATTAAAATTTTATTAGG | U-AACTCTACCCCTTTCTTTCATTC | GAAGGAGGGTTTAGTTATGT |
| MIR1247 | GTTTTTGGGTTTTTAGGTTAAGGTA | TCCCAAACCTTCTAATCCC | GTTTTTGGGTTTTTAGGTTAAGGTA | U-TCCCAAACCTTCTAATCCC | GTTTTTGAGTTTAAGTAGTTAATT |
| CDH13 | GYGAGGTGTTTATTTYGTATTTGT | AACCAACTTCCCAAATAAATCAAC | GYGAGGTGTTTATTTYGTATTTGT | U-AACCAACTTCCCAAATAAATCAAC | TGTTATGTAAAAYGAGGG |
| HAND2 | GGAGATTTTGTTGGGAAAATT | CAAAAATCTAAAACCCTAAATAAACC | GGAGATTTTGTTGGGAAAATT | U-CAAAAATCTAAAACCCTAAATAAACC | AAGTAGTTTAGTTTAGGAGAATTAT |
| HOXA4 | GGTTGGGGTTTAAAGTTTTTGT | AACCCCCCCACCAATAAA | GGTTGGGGTTTAAAGTTTTTGT | U-AACCCCCCCACCAATAAA | AGGGGTAGGATAGTAGTATTTA |
| SPOCK2 | TAGAGGGAGGAGAGTTGAGGATAG | TCCACCTAAAAAAATCTTAACTTCTACAATA | TAGAGGGAGGAGAGTTGAGGATAG | U-TCCACCTAAAAAAATCTTAACTTCTACAATA | GGAGGAGAGTTGAGGATAG |
| SOX17 | AGTTTTTTAGGGGGTAGGTGTAGT | CAAACCTTTTCTACACAAATATAACC | AGTTTTTTAGGGGGTAGGTGTAGT | U-CAAACCTTTTCTACACAAATATAACC | GGGGGAGGGGTAAG |
| GARA2 | GGGGTTAGGAGGAGGAGTAAGAGA | ACCACTCCCCAACCCTTCC | GGGGTTAGGAGGAGGAGTAAGAGA | U-ACCACTCCCCAACCCTTCC | GGAGAGGTTGTTTTTAGTAG |
| hTERT | TGTTGYGTAYGTGGGAAGTTT | AACCCTAAAACCCCAAAC | TGTTGYGTAYGTGGGAAGTTT | U-AACCCTAAAACCCCAAAC | TGGGAAGTTTTGGTTT |
| CDX1 | GTAGAGGAGGTTTTAGGGTTTAGTAT | CCAAACCCAAACTAACTAACCTA | GTAGAGGAGGTTTTAGGGTTTAGTAT | U-CCAAACCCAAACTAACTAACCTA | GTTATGTGTTGGATAAGGAT |
| SFRP1 | TGTTTTTTAAGGGGTGTTGA | CTCCRAAAACTACAAAACTAAAAT | TGTTTTTTAAGGGGTGTTGA | U-CTCCRAAAACTACAAAACTAAAAT | GTTTGGTTTTAGTAAAT |
| MGMT | TTGGTAAATTAAGGTATAGAGTTTT | AAACAATCTACGCATCCT | TTGGTAAATTAAGGTATAGAGTTTT | U-AAACAATCTACGCATCCT | GGAAGTTGGGAAGG |
| TWIST1 | GTTAAGTGAGGTGGGAAGGTTGA | CCCACCCCCTCAACAAAAC | GTTAAGTGAGGTGGGAAGGTTGA | U-CCCACCCCCTCAACAAAAC | GGAGAGGGGAGGAAA |
| MIR124A1 | AAAGGTGAAAGAAAGGAAGAGG | TCTCCCACTTCCACCCACA | GGGTGGGTAGAAGATGGAATAA | U-CCACCAAAAAAATACTATAATCCC | AATTAGGAAAAAGAAATAAA |
| MIR124A3 | GGGAGAAGTGTGGGTTTTTT | CCTTAATTATATAAACATTAAATCAAAATC | GGGAGGATTGGGATAGTAT | U-AACCTCCAAACCAAAAT | GGATTGGGATAGTATAG |
| MIR34BC | ATAGTTTAATTGTATTTGGAGAAA | ACCTTCCAAAAAAAATACCTAA | ATAGTTTAATTGTATTTGGAGAAA | U-ACCTTCCAAAAAAAATACCTAA | GGGTAGGTTGAGTAGGAT |
| ATP2B4 | GGGTTTTGTTGTAGTTTGAGAAT | ACTCCTTTAACTTAACCTTAAATCT | GGGTTTTGTTGTAGTTTGAGAAT | U-ACTCCTTTAACTTAACCTTAAATCT | TTTTGTTGTAGTTTGAGAATT |

U =biotin labeled universal primer tag: 5'-biotin-GGGACACCGCTGATCGTTTA
